# Do assortative preferences contribute to assortative mating for adiposity?

**DOI:** 10.1111/bjop.12055

**Published:** 2013-10-30

**Authors:** Claire I Fisher, Corey L Fincher, Amanda C Hahn, Anthony C Little, Lisa M DeBruine, Benedict C Jones

**Affiliations:** 1 Institute of Neuroscience and Psychology, University of GlasgowUK; 2 School of Natural Sciences, University of StirlingUK

## Abstract

Assortative mating for adiposity, whereby levels of adiposity in romantic partners tend to be positively correlated, has implications for population health due to the combined effects of partners' levels of adiposity on fertility and/or offspring health. Although assortative *preferences* for cues of adiposity, whereby leaner people are inherently more attracted to leaner individuals, have been proposed as a factor in assortative mating for adiposity, there have been no direct tests of this issue. Because of this, and because of recent work suggesting that facial cues of adiposity convey information about others' health that may be particularly important for mate preferences, we tested the contribution of assortative preferences for facial cues of adiposity to assortative mating for adiposity (assessed from body mass index, BMI) in a sample of romantic couples. Romantic partners' BMIs were positively correlated and this correlation was not due to the effects of age or relationship duration. However, although men and women with leaner partners showed stronger preferences for cues of low levels of adiposity, controlling for these preferences did not weaken the correlation between partners' BMIs. Indeed, own BMI and preferences were uncorrelated. These results suggest that assortative preferences for facial cues of adiposity contribute little (if at all) to assortative mating for adiposity.

## Background

Assortative mating for adiposity, whereby levels of adiposity in romantic partners tend to be positively correlated, has frequently been reported in studies of population health (see Di Castelnuovo, Quacquaruccio, Donati, De Gaetano, & Iacoviello, 2009 for a meta-analytic review). Moreover, this correlation between levels of adiposity in romantic partners appears to be robust (Di Castelnuovo *et al.*, 2009). For example, although correlations between levels of adiposity in romantic partners have typically been shown using body mass index (BMI; e.g., Jacobson, Torgerson, Sjostrom, & Bouchard, 2007; Silventoinen, Kaprio, Lahelma, Viken, & Rose, 2003), they have also been demonstrated using other measures of adiposity, including dual-energy X-ray absorptiometry (Speakman, Djafarian, Stewart, & Jackson, 2007) and skinfold thicknesses (Ginsburg, Livshits, Yakovenko, & Kobyliansky, 1999). Moreover, assortative mating for adiposity does not appear to be an artefact of potential confounds. For example, although age and adiposity tend to be positively correlated (e.g., Pasco, Nicholson, Brennan, & Kotowicz, 2012), as are romantic partners' ages (e.g., Watson *et al.*, 2004), assortative mating for adiposity is not simply a by-product of the combined effects of these correlations (e.g., Speakman *et al.*, 2007). Similarly, although socio-economic status and adiposity tend to be negatively correlated (e.g., Moore, Stunkard, & Srole, 1997) and romantic couples tend to be from similar social backgrounds (e.g., Schwartz & Mare, 2005; Smits, 2003), combined effects of these correlations do not fully explain assortative mating for adiposity (e.g., Silventoinen *et al.*, 2003). That the within-couples correlation between levels of adiposity appears to change very little as the duration of the relationship increases also suggests that assortative mating for adiposity is not simply due to the effects of the shared environment on adiposity (see Di Castelnuovo *et al.*, 2009 for a meta-analytic review).

Importantly, assortative mating for adiposity could have serious consequences for population health. For example, as there is a substantial genetic component to adiposity (reviewed in Speliotes *et al.*, 2010), assortative mating for adiposity may increase the proportion of individuals with high levels of adiposity (i.e., obese individuals) in the population (Hebebrand *et al.*, 2000; Speakman *et al.*, 2007). Indeed, some models of the effect of assortative mating for adiposity on the prevalence of obesity in a baseline population suggest that switching from random to completely assortative mating could more than double the percentage of obese individuals in the population within just two generations (Speakman *et al.*, 2007). The combined effects of men's and women's adiposity on the fertility of couples (i.e., the tendency for infertility to be particularly common in couples where *both* the man and woman have high levels of adiposity) may counteract, to some extent, this effect of assortative mating on rates of obesity (Ramlau-Hansen *et al.*, 2007). However, the combined effects of men's and women's adiposity on fertility also present additional evidence that assortative mating for adiposity can have negative effects on important aspects of population health (in this case, rates of infertility). In the light of findings such as these, many researchers have emphasized the importance of establishing why assortative mating for adiposity occurs (Courtiol, Picq, Godelle, Raymond, & Ferdy, 2010; Di Castelnuovo *et al.*, 2009; Hebebrand *et al.*, 2000; Speakman *et al.*, 2007).

Assortative *preferences* for cues of adiposity, whereby leaner people show stronger preferences for leaner individuals, are a potential explanation for the positive correlation between romantic partners' levels of adiposity (e.g., Courtiol *et al.*, 2010; Speakman *et al.*, 2007; Zietsch, Verweij, Heath, & Martin, 2011). Indeed, assortative preferences for other physical characteristics have been reported in several studies of human mate preferences (reviewed in, e.g., Havlicek & Roberts, 2009) and assortative preferences are thought to play a critical role in assortative mating for physical characteristics in several non-human species (e.g., Møller, 1994). Furthermore, men's and women's preferences for leaner body shapes in silhouettes of opposite-sex bodies are negatively correlated with their actual romantic partner's BMI (Courtiol *et al.*, 2010), although the cross-sectional design used in this work means that the causal direction of the relationship is unclear. Market-value-contingent preferences, whereby more attractive individuals demonstrate stronger preferences for attractive characteristics in images of potential mates (e.g., Little & Mannion, 2006), also suggest that assortative *preferences* for cues of adiposity could occur. However, while some work has suggested that preferences for cues of adiposity in body images are correlated with the perceiver's own BMI (Tovée, Emery, & Cohen-Tovée, 2000), this effect of own BMI on attractiveness judgements appears to be largely due to atypical perceptions in individuals with eating disorders (e.g., anorexia nervosa, Tovée *et al*., 2000). Indeed, some other studies also suggest that measures of own adiposity, including BMI, do not predict mating-related perceptions of body images, such as perceptions of potential mates' health or youth (Han, Morrison, & Lean, 1999) and attractiveness (Price, Pound, Dunn, Hopkins, & Kang, 2013). Although these results are not necessarily conclusive, findings such as these suggest that own adiposity may have little effect on body adiposity preferences, at least in healthy individuals (Speakman *et al.*, 2007), raising the possibility that assortative mating for adiposity is at least partly due to the additional constraints on the mate choices of individuals with higher levels of adiposity (see, e.g., Zietsch *et al.*, 2011).

Despite the fact that there have been no direct empirical tests of the role of assortative preferences in assortative mating for adiposity, the lack of evidence for a robust relationship between own adiposity and mating-related perceptions of bodies has led some researchers to conclude that assortative preferences contribute little to assortative mating for adiposity (e.g., Speakman *et al.*, 2007). However, focusing exclusively on *body* attractiveness may limit the conclusions that can be drawn about the role of mate preferences in assortative mating for adiposity. For example, although facial characteristics (including cues of adiposity) were obscured in the stimuli used in the above studies, *facial* cues of adiposity communicate information that is known to be important for human mate choice. For example, facial cues of adiposity communicate information about peoples' physical attractiveness (Coetzee, Perrett, & Stephen, 2009; Hume & Montgomerie, 2001), perceived health (Coetzee *et al.*, 2009), actual physical health (Coetzee *et al.*, 2009; Tinlin *et al.*, 2013), life expectancy (Reither, Hauser, & Swallen, 2009), immunocompetence (Rantala *et al.*, 2013), psychological condition (Tinlin *et al.*, 2013), and hormonal profile (Tinlin *et al.*, 2013). Indeed, perceived facial adiposity (i.e., the perception of fatness in the face) conveys information about health over and above that which is explained by BMI (Tinlin *et al.*, 2013) and some aspects of health (e.g., reported frequency and duration of respiratory infections) are more strongly correlated with facial adiposity than they are with BMI (Coetzee *et al.*, 2009). In addition, some studies suggest that facial cues are more important than body characteristics for judgements of men's and women's attractiveness, especially, but not exclusively, when bodies are clothed and when participants judged the attractiveness of potential mates for long-term relationships (e.g., Confer, Perilloux, & Buss, 2010; Currie & Little, 2009; Peters, Rhodes, & Simmons, 2007). Together, these results raise the intriguing possibility that individual differences in preferences for *facial* cues of adiposity may play an important role in assortative mating for adiposity. However, no study to date has addressed the relationships between preferences for facial cues of adiposity and either own or actual partner characteristics. Perhaps more importantly, it is also not known whether individual differences in preferences for facial cues of adiposity contribute to assortative mating for adiposity.

In the light of the above, we investigated the contribution of individual differences in preferences for cues of adiposity in opposite-sex faces to assortative mating for adiposity (measured using BMI). If controlling for the possible effects of individual differences in preferences for facial cues of adiposity weakens the positive correlation between romantic partners' BMIs, it would suggest that assortative preferences contribute to assortative mating for adiposity. However, if controlling for the possible effects of individual differences in preferences for cues of adiposity does not weaken the predicted correlation between romantic partners' BMIs, it would suggest that assortative preferences for facial cues of adiposity contribute little to assortative mating for adiposity. We assessed individual differences in preferences for facial cues of adiposity in two ways. One method measured participants' preferences for perceived facial adiposity. The other measured participants' preferences for facial characteristics associated with actual BMI. Given the correlations between perceived facial adiposity and measured BMI that were reported in previous studies (Coetzee *et al.*, 2009; Tinlin *et al.*, 2013), these two measures of adiposity preference were expected to be highly correlated.

## Methods

### Participants

Sixty-two heterosexual couples were recruited for the study. The mean age of the men was 21.8 years (*SD* = 1.96 years) and the mean age of the women was 21.2 years (*SD* = 1.94 years). The average duration of these couples' relationships was 18.4 months (*SD* = 15.1 months). Following Courtiol *et al*. (2010), the man and woman in each couple were tested at the same time, but were separated during testing.

### Stimuli

The stimuli that we used to assess preferences for cues of adiposity in opposite-sex faces were full-colour images of 50 white men (mean age = 24.2 years, *SD* = 3.99 years) and 50 white women (mean age = 24.3 years, *SD* = 4.01 years). All images were taken under standardized lighting conditions and against a constant background. All individuals photographed posed with neutral expressions and direct gaze. Images were standardized on pupil position and masked so that clothing was not visible. Height and weight measurements were taken from each of these 50 men (mean height = 180.2 cm, *SD* = 6.62 cm; mean weight = 77.3 kg, *SD* = 12.4 kg) and 50 women (mean height = 168.6 cm, *SD* = 6.48 cm; mean weight = 57.2 kg, *SD* = 11.4 kg).

The height and weight measurements were used to calculate each of the photographed individuals' BMI (men: *M* = 23.7 kg/m^2^, *SD* = 3.13 kg/m^2^, range = 17.7–31.0 kg/m^2^; women: *M* = 20.1 kg/m^2^, *SD* = 3.66 kg/m^2^, range = 16.2–38.4 kg/m^2^). According to the World Health Organization's (WHO) classifications (World Health Organization, 2000), 28% of the women and 2% of the men were in the underweight BMI category (<18.5 kg/m^2^), 68% of the women and 68% of the men were in the normal category (18.5–24.99 kg/m^2^), and 4% of the women and 30% of the men were in the overweight category (>25 kg/m^2^). None of these individuals were extremely underweight (i.e., none had BMI < 15 kg/m^2^, Bray, 1978) and only two individuals (both women) had BMI < 17 kg/m^2^.

Methods for collecting ratings of facial adiposity were identical to those used in previous studies (Coetzee *et al.*, 2009; Tinlin *et al.*, 2013). The 50 male face images were rated for adiposity using a 1 (*very underweight*) to 7 (*very overweight*) scale by 60 heterosexual raters (30 women, 30 men; mean age = 22.08 years, *SD* = 3.53 years). The order in which images were presented was fully randomized. A different group of 60 heterosexual raters (30 women, 30 men; mean age = 23.18 years, *SD* = 3.04 years) rated the 50 female images for adiposity using the same scale. Inter-rater agreement was extremely high for adiposity ratings of both men's (Cronbach's alpha = .98) and women's (Cronbach's alpha = .98) faces. Consequently, we calculated the average adiposity rating for each face image (male faces: *M* = 3.73, *SD* = 0.76; female faces: *M* = 3.79, *SD* = 0.80). Men's and women's average ratings were highly correlated for both men's faces (*r* = .97, *N* = 50, *p* < .001) and women's faces (*r* = .96, *N* = 50, *p* < .001). Consistent with prior work (Coetzee *et al.*, 2009; Tinlin *et al.*, 2013), BMI and rated facial adiposity were positively correlated (men: *r* = .55, *N* = 50, *p* < .001; women: *r* = .69, *N* = 50, *p* < .001).

### Procedure

Height and weight measurements were taken from each of the 62 men (mean height = 180.6 cm, *SD* = 6.55 cm; mean weight = 77.1 kg, *SD* = 11.2 kg) and 62 women (mean height = 166.3 cm, *SD* = 5.35 cm; mean weight = 64.0 kg, *SD* = 12.6 kg) who made up our romantic couples. These measurements were used to calculate BMI (men: *M* = 23.6 kg/m^2^, *SD* = 3.13 kg/m^2^, range = 16.4–31.7 kg/m^2^; women: *M* = 23.1 kg/m^2^, *SD* = 4.19 kg/m^2^, range = 18.0–37.1 kg/m^2^). According to the World Health Organization (2000) classifications, 5% of the women and 5% of the men were in the underweight BMI category (<18.5 kg/m^2^), 72% of the women and 69% of the men were in the normal category (18.5–24.99 kg/m^2^), and 23% of the women and 26% of the men were in the overweight category (>25 kg/m^2^). None of these individuals were extremely underweight (i.e., none had BMI < 15 kg/m^2^, Bray, 1978) and only one individual (a man) had BMI < 17 kg/m^2^.

Each of the 62 men in our study rated the attractiveness of the 50 female faces described in our Stimuli section. In addition, each of the 62 women in our study rated the attractiveness of the 50 male faces described in our Stimuli section. Attractiveness ratings were made using a 1 (*much less attractive than average*) to 7 (*much more attractive than average*) scale. Following previous studies of preferences for facial cues of adiposity (e.g., Coetzee *et al.*, 2009), the order in which images were presented was fully randomized and each image remained on screen until the participant had entered their rating. Inter-rater agreement, as measured by Cronbach's alpha, was high for both sets of ratings (both >.96).

## Results

### Calculating preference scores

For each couple, we calculated the Pearson product-moment correlation between (1) the woman's attractiveness rating for each of the 50 men's faces and those 50 men's rated facial adiposity (mean *r* = −.16, *SD* = .17, *p* = .27); (2) the woman's attractiveness rating for each of the 50 men's faces and those 50 men's BMI (mean *r* = −.13, *SD* = .13, *p* = .37); (3) the man's attractiveness rating for each of the 50 women's faces and those 50 women's rated facial adiposity (mean *r* = −.23, *SD* = .14, *p* = .11); and (4) the man's attractiveness rating for each of the 50 women's faces and those 50 women's BMI (mean *r* = −.27, *SD* = .10, *p* = .058).2010 Note that this procedure produces two correlation coefficients for each participant (one being a measure of their preference for perceived facial adiposity and the other a measure of their preference for facial cues of BMI). These correlation coefficient scores served as the dependent variables in subsequent analyses. For each of these preference scores, larger positive values indicate stronger preferences for facial characteristics associated with higher BMI and larger negative values indicate stronger preferences for facial characteristics associated with lower BMI. This method for calculating preference scores by deriving the correlation coefficients for the relationships between each participant's attractiveness ratings of individual faces and another variable (in this study, either rated facial adiposity or BMI) has been used in previous studies to assess individual differences in face preferences (e.g., Fisher, Fincher, Hahn, DeBruine, & Jones, 2013).

Next, we analysed women's preference scores (i.e., their preferences for perceived facial adiposity and their preferences for facial cues of BMI) using factor analysis. This analysis produced a single factor that explained 87% of the variance in women's preference scores and was highly correlated with both of the original variables (both *r* = .93). We labelled this factor *women's preference for cues of BMI in men's faces*. A corresponding analysis for men's preference scores also produced a single factor. This factor explained 89% of the variance in men's preference scores and was highly correlated with both of the original variables (both *r* = .94). We labelled this factor *men's preference for cues of BMI in women's faces*. On both of these factors, higher scores indicate stronger preferences for facial characteristics associated with higher BMI. Romantic partners' preferences for cues of BMI in opposite-sex faces were positively correlated (*r* = .31, *N* = 62, *p* = .016).

### Assortative mating for BMI in our sample

We first tested for evidence of assortative mating for BMI in our sample. As predicted, romantic partners' BMIs were positively correlated (*r* = .49, *N* = 62, *p* < .001). Subsequent partial correlation analyses showed that this correlation between romantic partners' BMIs remained significant when we controlled for the possible effects of women's age (partial *r* = .38, *p* = .003), men's age (partial *r* = .45, *p* < .001), or both men's age and women's age simultaneously (partial *r* = .38, *p* = .003). These results show that assortative mating for BMI in this sample is not due to the combined effects of older individuals tending to have higher BMI and couples tending to be similar in age. Similarly, results for a partial analysis controlling for the possible effects of relationship duration (partial *r* = .45, *p* < .001) suggested that relationship duration had little effect on the strength of assortative mating for BMI in this sample.

### Assortative preferences and assortative mating for BMI

To investigate whether individual differences in preferences for cues of BMI in opposite-sex faces contributed to assortative mating for BMI, we conducted a second set of partial correlation analyses. These analyses showed that the correlation between romantic partners' BMIs (*r* = .49, *N* = 62, *p* < .001) changed very little when we controlled for the possible effects of women's preferences for cues of BMI in men's faces (partial *r* = .50, *p* < .001), men's preferences for cues of BMI in women's faces (partial *r* = .47, *p* < .001), both men's and women's preferences for cues of BMI in opposite-sex faces simultaneously (partial *r* = .50, *p* < .001), or the average of men's and women's preferences for cues of BMI in opposite-sex faces (partial *r* = .47, *p* < .001). These results suggest that individual differences in preferences for facial cues of BMI contributed little (if at all) to assortative mating for BMI.

### Preferences for cues of BMI and own/partner's BMI

Although our results suggest that individual differences in preferences for facial cues of BMI contribute little to assortative mating for BMI, it is still possible that participants' preferences for cues of BMI in opposite-sex faces are correlated with either their own BMI or their partner's BMI. Thus, we investigated the intercorrelations among men's BMI, women's BMI, men's preferences for cues of BMI in women's faces, and women's preferences for cues of BMI in men's faces (Table 2010). Men's and women's preferences for cues of BMI in opposite-sex faces predicted their romantic partner's BMI (men's preferences: *r* = .30, *N* = 62, *p* = .017; women's preferences: *r* = .30, *N* = 62, *p* = .017), indicating the individuals who showed stronger preferences for cues of low BMI in opposite-sex faces had leaner romantic partners. In contrast with these results for partner's BMI, own BMI predicted neither men's nor women's preferences for facial cues of BMI (men's preferences: *r* = .17, *N* = 62, *p* = .19; women's preferences: *r* = .04, *N* = 62, *p* = .77).

**Table 1:** Correlations among men's body mass index (BMI), women's BMI, men's preferences for cues of BMI in women's faces, and women's preferences for cues of BMI in men's faces

	Women's BMI	Men's preference for cues of BMI	Women's preference for cues of BMI
Men's BMI	.49**	.17^NS^	.30*
Women's BMI		.30*	.04^NS^
Men's preference for cues of BMI			.31*

Note *N* = 62 for each correlation

NS *p* > .05

* *p* < .05

** *p* < .001 (all non-significant correlations were *p* > .19).

Finally, we conducted regression analyses to test whether preferences for cues of BMI in opposite-sex faces and own BMI *independently* predicted actual romantic partner's BMI. Partner's BMI was entered as the dependent variable and own BMI and preferences for cues of BMI were entered simultaneously as predictors. Separate regression analyses were conducted for men and women. For men, their own BMI (*t* = 4.04, standardized beta = .45, *p* < .001) and their preference for cues of BMI in women's faces (*t* = 2.03, standardized beta = .23, *p* = .047) each independently predicted their romantic partner's BMI. This pattern was also observed for women; own BMI (*t* = 4.43, standardized beta = .48, *p* < .001) and preference for cues of BMI in men's faces (*t* = 2.63, standardized beta = .28, *p* = .011) each independently predicted their romantic partner's BMI. Stepwise versions of these analyses showed that adding preferences to a model in which only own BMI was a predictor significantly increased the variance in partner BMI explained by the model (men's preferences: *R*^2^ change = .05, *p* = .047; women's preferences: *R*^2^ change = .08, *p* = .011).

## Discussion

As in many previous studies that have reported assortative mating for adiposity (see Di Castelnuovo *et al.*, 2009 for a meta-analytic review), romantic partners' BMIs were positively correlated. Partial correlation analyses showed that this correlation between romantic partners' BMIs was not due to the possible effects of age or relationship duration (see also, e.g., Di Castelnuovo *et al.*, 2009; Speakman *et al.*, 2007).

Consistent with previous work showing that men's and women's preferences for leaner *body* images were correlated with their actual romantic partner's BMI (Courtiol *et al.*, 2010), men's and women's preferences for facial cues of BMI in opposite-sex faces were correlated with their romantic partners' BMIs; people with leaner partners showed stronger preferences for cues of low BMI in opposite-sex faces. In addition, romantic partners' preferences for cues of BMI in opposite-sex faces were concordant; the romantic partners of people who had particularly strong preferences for cues of low BMI also tended to demonstrate stronger preferences for these facial cues. Despite these significant correlations between BMI preferences and partner's BMI and between romantic partners' BMI preferences, we found no evidence that individual differences in preferences for facial cues of BMI contributed to assortative mating for BMI. Indeed, controlling for the possible effects of assortative preferences for cues of BMI had no discernible effect on the correlation between romantic partners' BMIs. Additional analyses indicated that this pattern of results was due to the independence of own BMI and preferences for facial cues of BMI; own BMI and preference for facial cues of BMI were not significantly correlated in our sample and independently predicted partner's BMI in both men and women. In other words, although preferences for cues of BMI in opposite-sex faces explained some of the variance in the adiposity of men's and women's romantic partners, this variance was wholly independent of that which was explained by assortative mating for BMI. Together, these results then suggest that *assortative preferences* contribute little (if at all) to *assortative mating* for adiposity. That own BMI and preference for facial cues of BMI were not significantly correlated in our sample is consistent with other recent work that observed no significant correlations between measures of participants' own adiposity and their preferences for cues of adiposity in opposite-sex bodies (Price *et al.*, 2013).

Although factors not considered in this study will almost certainly contribute to assortative mating for adiposity (e.g., social homogamy, Silventoinen *et al.*, 2003), our results are consistent with the proposal that assortative mating for adiposity is not due to assortative preferences for cues of adiposity, but is likely to be due (at least in part) to the additional constraints on the mate choices of individuals with higher levels of adiposity (Speakman *et al.*, 2007; see also Zietsch *et al.*, 2011). Additional constraints on the mate choices of individuals with higher levels of adiposity may arise because the pool of people who are willing to choose mates with higher levels of adiposity will be smaller (and include a higher proportion of fatter individuals) than the pool of people who are willing to choose relatively lean mates (Speakman *et al.*, 2007). Indeed, individuals with higher levels of adiposity do report having had fewer sexual partners in the previous year, consistent with the proposal that their mate choices are more constrained (e.g., Bajos, Wellings, Laborde, & Moreau, 2010). Our results are also consistent with recent research on the genetic basis of assortative mating for BMI, which suggests that it is more likely to be due to the heritability of BMI than heritability of preferences for cues of BMI (Zietsch *et al.*, 2011).

Findings for attractiveness judgements of opposite-sex faces are often assumed to give insight into the factors that influence human mate choice (for reviews see Gangestad & Thornhill, 2008; Little, Jones, & DeBruine, 2011; Rhodes, 2006) and are frequently interpreted as evidence that sexual selection has been an important factor in the evolution of human face preferences (Gangestad & Thornhill, 2008; Little *et al.*, 2011; Rhodes, 2006). However, many researchers have noted that few studies have investigated the possible correlations between face preferences and actual partner choice (e.g., Penton-Voak, 2011; Puts, Jones, & DeBruine, 2012). Our data show that preferences are linked to real-life mate choice as they demonstrate correlations between partner BMI and both men's and women's face preferences, revealing a pathway through which sexual selection could have influenced preferences for facial cues of adiposity. Although the conclusions that can be drawn on this point from our data are limited to conclusions relating to preferences for facial cues of adiposity, similar tests involving preferences for other facial characteristics (e.g., sex-stereotypical shape cues) may provide converging evidence for links between face preferences and romantic partner choice (see, e.g., DeBruine, Fincher, Watkins, Little, & Jones, 2012).

There are several limitations to this study that should be acknowledged. First, the cross-sectional design of this study means that the causal direction of the relationship between face preferences and partner BMI is unclear. Studies using longitudinal designs to investigate this issue may clarify whether mate preferences directly influence mate choices, mate choices directly influence mate preferences, or both. Second, the ranges of BMIs represented in our stimuli and our participant couples were relatively narrow (e.g., did not include many obese individuals). Further studies with a greater proportion of overweight and obese individuals could yet implicate assortative preferences in assortative mating for adiposity. Such studies could also use face stimuli that were more closely matched in age to the participants than was the case in this study where the faces were, on average, between 2 and 3 years older than the participants. Third, we used a subjective measure of facial adiposity that, although positively correlated with actual BMI, may still be subject to perceptual biases (e.g., attractiveness halo effects, Dion, Berscheid, & Walster, 1972). Subsequent studies exploring individual differences in preferences for facial cues of BMI may consider employing more objective measures of facial adiposity. However, although some facial metric measures of adiposity have been developed that correlate reasonably well with actual BMI (see Coetzee, Chen, Perrett, & Stephen, 2010), these measures can be subject to systematic errors (see Schneider, Hecht, & Carbon, 2012). Further work is needed to develop more robust, objective measures of facial adiposity. Fourth, although our analyses reveal individual differences in preferences for facial correlates of BMI, it is unclear whether these individual differences reflect variation in motivation to obtain mates with low BMI or variation in motivation to obtain mates who, for example, lead particular lifestyles or possess particular hormonal profiles that are correlated with BMI. Studies investigating the determinants of individual differences in preferences for cues of BMI may clarify this issue (see, e.g., Fisher *et al.*, 2013).

This study directly tested the contribution of assortative preferences for cues of BMI in opposite-sex faces to assortative mating for adiposity in a sample of romantic couples. Analyses suggested that individual differences in preferences for facial cues of BMI contribute little (if at all) to assortative mating for BMI. However, both men's and women's preferences for facial cues of BMI were positively correlated with their actual romantic partners' BMIs. Thus, our data potentially not only implicate preferences for cues of BMI in partner choices but also show that partner choice is not redundant with face preferences, at least with regard to preferences for cues of BMI. Indeed, some differences between preferences for cues of BMI and actual mate choices would be expected, given that mate choices are likely to be constrained in ways that mate preferences are not. Importantly, the causal direction of the relationship between preferences for facial cues of BMI and partner BMI is unclear. One possibility is that preferences for cues of adiposity directly contribute to partner selection (i.e., preferring cues associated with lower BMI causes people to choose leaner partners). Another possibility is that having a leaner partner causes people to prefer cues of lower levels of facial adiposity. For example, people may realign their preferences to match partner characteristics to reduce cognitive dissonance and experiments have demonstrated that increasing participants' recent visual experience with images of the bodies of individuals with higher levels of adiposity increases their preferences for facial characteristics that are correlated with higher BMIs (Re *et al.*, 2011). That these possibilities are by no means mutually exclusive may have important implications for the mechanisms and processes through which individuals' mate preferences develop. For example, people typically have more than one romantic partner in the course of their lives (e.g., Brown & Sinclair, 1999). Consequently, if current partner choice influences preferences, these preferences may influence future partner choice, establishing a feedback loop that amplifies the effects of early mate choices on partner choice later in life. Consistent with this proposal, recent work has found that characteristics of participants' sexual experiences that occurred early in adulthood predicted their physical and emotional satisfaction with their current sexual interactions, even when the effects of global sexual satisfaction were controlled for statistically (Smith & Shaffer, 2013). Although work on the development of human mate preferences has typically focused on experiences in early life (e.g., imprinting-like effects in childhood, Perrett *et al.*, 2002), the possibility that early mate choices are another important factor for the development of mate preferences has received relatively little attention. We suggest that studies directly testing the role of previous mate choices in future mate choice and mate preferences may provide important insights into the ontogeny of mating behaviour.
